# Dichlorido{2-(morpholin-4-yl)-*N*-[1-(pyridin-2-yl)ethyl­idene]ethanamine-κ^3^ *N*,*N*′,*N*′′}copper(II) monohydrate

**DOI:** 10.1107/S1600536811004892

**Published:** 2011-02-12

**Authors:** Nura Suleiman Gwaram, Hamid Khaledi, Hapipah Mohd Ali

**Affiliations:** a Department of Chemistry, University of Malaya, 50603 Kuala Lumpur, Malaysia

## Abstract

In the title compound, [CuCl_2_(C_13_H_19_N_3_O)]·H_2_O, the tridentate Schiff base ligand and the two Cl atoms complete a distorted square-pyramidal coordination geometry around the Cu^II^ ion in which the three N atoms and one Cl atom are located in the basal plane and the other Cl atom is at the apical position. In the crystal, O—H⋯Cl hydrogen bonds link the complex mol­ecules and the uncoordinated water mol­ecules into infinite chains along the *a* axis. The chains are further connected into a three-dimensional network *via* C—H⋯O and C—H⋯Cl inter­actions.

## Related literature

For the structures of CuCl_2_ complexes with similar ligands, see: Saleh Salga *et al.* (2010 ▶); Wang *et al.* (2009 ▶). For the structure of a CdCl_2_ complex with the same Schiff base ligand, see: Ikmal Hisham *et al.* (2010 ▶). For a description of the geometry of complexes with a five-coordinate metal atom, see: Addison *et al.* (1984 ▶).
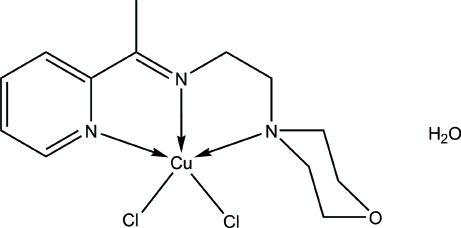


## Experimental

### 

#### Crystal data

[CuCl_2_(C_13_H_19_N_3_O)]·H_2_O
*M* *_r_* = 385.77
Monoclinic, 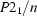

*a* = 7.9194 (8) Å
*b* = 8.5793 (8) Å
*c* = 22.925 (2) Å
β = 91.981 (1)°
*V* = 1556.6 (3) Å^3^
*Z* = 4
Mo *K*α radiation
μ = 1.75 mm^−1^
*T* = 100 K
0.18 × 0.16 × 0.09 mm

#### Data collection

Bruker APEXII CCD diffractometer
Absorption correction: multi-scan (*SADABS*; Sheldrick, 1996 ▶) *T*_min_ = 0.743, *T*_max_ = 0.858
9634 measured reflections
3348 independent reflections
2948 reflections with *I* > 2σ(*I*)
*R*_int_ = 0.023

#### Refinement

*R*[*F* ^2^ > 2σ(*F* ^2^)] = 0.025
*wR*(*F* ^2^) = 0.060
*S* = 1.05
3348 reflections
197 parameters
2 restraints
H atoms treated by a mixture of independent and constrained refinement
Δρ_max_ = 0.37 e Å^−3^
Δρ_min_ = −0.34 e Å^−3^

### 

Data collection: *APEX2* (Bruker, 2007 ▶); cell refinement: *SAINT* (Bruker, 2007 ▶); data reduction: *SAINT*; program(s) used to solve structure: *SHELXS97* (Sheldrick, 2008 ▶); program(s) used to refine structure: *SHELXL97* (Sheldrick, 2008 ▶); molecular graphics: *X-SEED* (Barbour, 2001) ▶; software used to prepare material for publication: *SHELXL97* and *publCIF* (Westrip, 2010 ▶).

## Supplementary Material

Crystal structure: contains datablocks I, global. DOI: 10.1107/S1600536811004892/is2674sup1.cif

Structure factors: contains datablocks I. DOI: 10.1107/S1600536811004892/is2674Isup2.hkl

Additional supplementary materials:  crystallographic information; 3D view; checkCIF report

## Figures and Tables

**Table 1:** Hydrogen-bond geometry (Å, °)

*D*—H⋯*A*	*D*—H	H⋯*A*	*D*⋯*A*	*D*—H⋯*A*
O2—H2*A*⋯Cl2^i^	0.84 (2)	2.35 (2)	3.1829 (16)	173 (2)
O2—H2*B*⋯Cl1	0.83 (2)	2.48 (2)	3.2841 (18)	164 (2)
C2—H2⋯O2^ii^	0.95	2.41	3.307 (2)	156
C3—H3⋯Cl2^iii^	0.95	2.82	3.619 (2)	142
C4—H4⋯O2^iv^	0.95	2.50	3.445 (2)	172
C7—H7*A*⋯Cl1^v^	0.98	2.68	3.6179 (19)	161
C8—H8*A*⋯O1^vi^	0.99	2.47	3.336 (2)	146
C10—H10*B*⋯Cl1	0.99	2.79	3.4496 (19)	124
C10—H10*A*⋯Cl2	0.99	2.71	3.3566 (19)	123

Symmetry codes: (i) 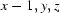; (ii) 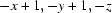; (iii) 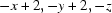; (iv) 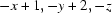; (v) 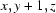; (vi) 
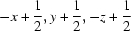
.

**Fig. 1.**
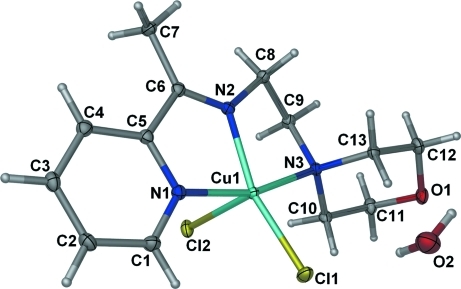
Displacement ellipsoid plot of the title compound at the 50% probability level. Hydrogen atoms are drawn as spheres of arbitrary radii.

## supplementary crystallographic information

### Comment

The asymmetric unit of the title compound consists of a copper(II) complex and one molecule of water. Like the CdCl_2_ complex of the Schiff base, 2-morpholino-*N*-[1-(2-pyridyl)ethylidene]ethanamine, (Ikmal Hisham *et al.*, 2010) the metal ion in the present structure is five-coordinated by the *N,N',N"*-tridentate Schiff base ligand and two Cl atoms in a distorted square-pyramidal geometry, the τ value (Addison *et al.*,1984) being 0.15. The Cu—Cl and Cu—N interatomic distances are comparable to the values reported in the literature (Saleh Salga *et al.*, 2010; Wang *et al.*, 2009). In the crystal, the adjacent metal complexes and water molecules are linked into a three-dimensional network *via* O—H···Cl, C—H···Cl and C—H···O interactions. In addition, intramolecular C—H···Cl hydrogen bonding is observed.

### Experimental

A mixture of 2-acetylpyridine (0.20 g, 1.65 mmol) and 4-(2-aminoethyl)morpholine (0.21 g, 1.65 mmol) in ethanol (20 ml) was refluxed. After 2 hr a solution of copper(II) chloride dihydrate (0.28 g, 1.65 mmol) in a minimum amount of ethanol was added and the resulting solution was refluxed for 30 min, then set aside at room temperature. The crystals of the title complex were obtained after a few days.

### Refinement

The C-bound hydrogen atoms were placed at calculated positions (C—H 0.95–0.99 Å) and were treated as riding on their parent atoms. The O-bound H atoms were placed in a difference Fourier map, and were refined with distance restraint of O—H 0.84 (2) Å. For all hydrogen atoms *U*_iso_(H) were set to 1.2–1.5 times *U*_eq_(carrier atom).

### Crystal data

**Table:**

[CuCl_2_(C_13_H_19_N_3_O)]·H_2_O	*F*(000) = 796
*M**_r_* = 385.77	*D*_x_ = 1.646 Mg m^−^^3^
Monoclinic, *P*2_1_/*n*	Mo *K*α radiation, λ = 0.71073 Å
Hall symbol: -P 2yn	Cell parameters from 4229 reflections
*a* = 7.9194 (8) Å	θ = 2.5–28.2°
*b* = 8.5793 (8) Å	µ = 1.75 mm^−^^1^
*c* = 22.925 (2) Å	*T* = 100 K
β = 91.981 (1)°	Block, green
*V* = 1556.6 (3) Å^3^	0.18 × 0.16 × 0.09 mm
*Z* = 4	

### Data collection

**Table:**

Bruker APEXII CCD diffractometer	3348 independent reflections
Radiation source: fine-focus sealed tube	2948 reflections with *I* > 2σ(*I*)
graphite	*R*_int_ = 0.023
φ and ω scans	θ_max_ = 27.0°, θ_min_ = 2.5°
Absorption correction: multi-scan (*SADABS*; Sheldrick, 1996)	*h* = −10→10
*T*_min_ = 0.743, *T*_max_ = 0.858	*k* = −10→10
9634 measured reflections	*l* = −29→28

### Refinement

**Table:**

Refinement on *F*^2^	Primary atom site location: structure-invariant direct methods
Least-squares matrix: full	Secondary atom site location: difference Fourier map
*R*[*F*^2^ > 2σ(*F*^2^)] = 0.025	Hydrogen site location: inferred from neighbouring sites
*wR*(*F*^2^) = 0.060	H atoms treated by a mixture of independent and constrained refinement
*S* = 1.05	*w* = 1/[σ^2^(*F*_o_^2^) + (0.0258*P*)^2^ + 0.893*P*] where *P* = (*F*_o_^2^ + 2*F*_c_^2^)/3
3348 reflections	(Δ/σ)_max_ = 0.001
197 parameters	Δρ_max_ = 0.37 e Å^−^^3^
2 restraints	Δρ_min_ = −0.34 e Å^−^^3^

### Special details

**Table:**

Geometry. All e.s.d.'s (except the e.s.d. in the dihedral angle between two l.s. planes) are estimated using the full covariance matrix. The cell e.s.d.'s are taken into account individually in the estimation of e.s.d.'s in distances, angles and torsion angles; correlations between e.s.d.'s in cell parameters are only used when they are defined by crystal symmetry. An approximate (isotropic) treatment of cell e.s.d.'s is used for estimating e.s.d.'s involving l.s. planes.
Refinement. Refinement of *F*^2^ against ALL reflections. The weighted *R*-factor *wR* and goodness of fit *S* are based on *F*^2^, conventional *R*-factors *R* are based on *F*, with *F* set to zero for negative *F*^2^. The threshold expression of *F*^2^ > σ(*F*^2^) is used only for calculating *R*-factors(gt) *etc*. and is not relevant to the choice of reflections for refinement. *R*-factors based on *F*^2^ are statistically about twice as large as those based on *F*, and *R*- factors based on ALL data will be even larger.

### Fractional atomic coordinates and isotropic or equivalent isotropic displacement parameters (Å^2^)

**Table:**

	*x*	*y*	*z*	*U*_iso_*/*U*_eq_	
Cu1	0.60251 (3)	0.86379 (2)	0.122328 (9)	0.01099 (7)	
Cl1	0.49087 (6)	0.62132 (5)	0.11931 (2)	0.01873 (11)	
Cl2	0.90458 (6)	0.85029 (5)	0.15951 (2)	0.01773 (11)	
O1	0.32231 (17)	0.82636 (16)	0.30317 (6)	0.0183 (3)	
N1	0.68558 (19)	0.86674 (17)	0.03959 (7)	0.0129 (3)	
N2	0.60269 (19)	1.09006 (17)	0.10733 (7)	0.0119 (3)	
N3	0.51025 (18)	0.92685 (17)	0.20213 (6)	0.0114 (3)	
C1	0.7350 (2)	0.7448 (2)	0.00826 (8)	0.0159 (4)	
H1	0.7128	0.6427	0.0221	0.019*	
C2	0.8177 (2)	0.7617 (2)	−0.04381 (8)	0.0167 (4)	
H2	0.8513	0.6729	−0.0653	0.020*	
C3	0.8501 (2)	0.9106 (2)	−0.06373 (8)	0.0156 (4)	
H3	0.9077	0.9254	−0.0990	0.019*	
C4	0.7975 (2)	1.0386 (2)	−0.03157 (8)	0.0147 (4)	
H4	0.8168	1.1417	−0.0449	0.018*	
C5	0.7169 (2)	1.0126 (2)	0.01989 (8)	0.0121 (4)	
C6	0.6645 (2)	1.1396 (2)	0.05987 (8)	0.0127 (4)	
C7	0.6910 (2)	1.3055 (2)	0.04320 (8)	0.0172 (4)	
H7A	0.6395	1.3737	0.0719	0.026*	
H7B	0.6385	1.3247	0.0045	0.026*	
H7C	0.8124	1.3271	0.0422	0.026*	
C8	0.5483 (2)	1.1856 (2)	0.15617 (8)	0.0127 (4)	
H8A	0.4272	1.2127	0.1510	0.015*	
H8B	0.6148	1.2832	0.1587	0.015*	
C9	0.5779 (2)	1.0881 (2)	0.21117 (8)	0.0132 (4)	
H9A	0.7005	1.0830	0.2210	0.016*	
H9B	0.5212	1.1379	0.2442	0.016*	
C10	0.5778 (2)	0.8179 (2)	0.24813 (8)	0.0139 (4)	
H10A	0.7017	0.8316	0.2522	0.017*	
H10B	0.5557	0.7094	0.2353	0.017*	
C11	0.5017 (2)	0.8419 (2)	0.30720 (8)	0.0174 (4)	
H11A	0.5492	0.7642	0.3352	0.021*	
H11B	0.5316	0.9471	0.3221	0.021*	
C12	0.2547 (2)	0.9419 (2)	0.26393 (8)	0.0171 (4)	
H12A	0.2853	1.0468	0.2789	0.021*	
H12B	0.1299	0.9340	0.2620	0.021*	
C13	0.3217 (2)	0.9220 (2)	0.20293 (8)	0.0130 (4)	
H13A	0.2818	0.8210	0.1867	0.016*	
H13B	0.2749	1.0057	0.1774	0.016*	
O2	0.0878 (2)	0.59912 (18)	0.08146 (7)	0.0273 (3)	
H2A	0.035 (3)	0.659 (3)	0.1032 (10)	0.033*	
H2B	0.183 (2)	0.602 (3)	0.0978 (11)	0.033*	

### Atomic displacement parameters (Å^2^)

**Table:**

	*U*^11^	*U*^22^	*U*^33^	*U*^12^	*U*^13^	*U*^23^
Cu1	0.01348 (12)	0.00927 (11)	0.01044 (12)	0.00023 (8)	0.00364 (8)	0.00007 (8)
Cl1	0.0256 (3)	0.0112 (2)	0.0200 (2)	−0.00334 (17)	0.00966 (19)	−0.00245 (17)
Cl2	0.0123 (2)	0.0245 (2)	0.0165 (2)	0.00191 (17)	0.00273 (17)	0.00437 (18)
O1	0.0157 (7)	0.0231 (7)	0.0165 (7)	−0.0002 (5)	0.0050 (5)	0.0047 (6)
N1	0.0131 (7)	0.0133 (7)	0.0124 (8)	0.0007 (6)	0.0013 (6)	0.0005 (6)
N2	0.0124 (7)	0.0112 (7)	0.0120 (8)	0.0006 (6)	0.0008 (6)	−0.0010 (6)
N3	0.0109 (7)	0.0114 (7)	0.0120 (8)	−0.0005 (6)	0.0013 (6)	−0.0006 (6)
C1	0.0190 (10)	0.0125 (9)	0.0162 (10)	0.0001 (7)	0.0019 (8)	−0.0010 (7)
C2	0.0175 (9)	0.0177 (9)	0.0151 (10)	0.0032 (7)	0.0029 (7)	−0.0031 (7)
C3	0.0142 (9)	0.0215 (10)	0.0113 (9)	0.0014 (7)	0.0017 (7)	0.0002 (7)
C4	0.0145 (9)	0.0155 (9)	0.0142 (9)	0.0001 (7)	0.0004 (7)	0.0017 (7)
C5	0.0127 (9)	0.0130 (8)	0.0107 (9)	0.0010 (7)	0.0003 (7)	−0.0004 (7)
C6	0.0108 (8)	0.0141 (9)	0.0130 (9)	0.0002 (7)	0.0003 (7)	0.0008 (7)
C7	0.0229 (10)	0.0124 (9)	0.0169 (10)	0.0016 (7)	0.0070 (8)	0.0016 (7)
C8	0.0150 (9)	0.0109 (8)	0.0124 (9)	−0.0008 (7)	0.0031 (7)	−0.0010 (7)
C9	0.0145 (9)	0.0126 (9)	0.0126 (9)	−0.0025 (7)	0.0007 (7)	−0.0029 (7)
C10	0.0127 (9)	0.0155 (9)	0.0134 (9)	0.0024 (7)	0.0007 (7)	0.0033 (7)
C11	0.0160 (9)	0.0228 (10)	0.0134 (9)	0.0002 (8)	0.0021 (7)	0.0025 (8)
C12	0.0152 (9)	0.0194 (9)	0.0171 (10)	0.0013 (7)	0.0056 (8)	−0.0004 (8)
C13	0.0099 (8)	0.0144 (9)	0.0148 (9)	−0.0005 (7)	0.0009 (7)	0.0000 (7)
O2	0.0307 (9)	0.0231 (8)	0.0285 (9)	0.0035 (7)	0.0053 (7)	−0.0013 (7)

### Geometric parameters (Å, °)

**Table:**

Cu1—N2	1.9715 (15)	C5—C6	1.491 (2)
Cu1—N1	2.0290 (15)	C6—C7	1.490 (2)
Cu1—N3	2.0654 (15)	C7—H7A	0.9800
Cu1—Cl1	2.2604 (5)	C7—H7B	0.9800
Cu1—Cl2	2.5143 (5)	C7—H7C	0.9800
O1—C11	1.427 (2)	C8—C9	1.524 (2)
O1—C12	1.430 (2)	C8—H8A	0.9900
N1—C1	1.336 (2)	C8—H8B	0.9900
N1—C5	1.356 (2)	C9—H9A	0.9900
N2—C6	1.281 (2)	C9—H9B	0.9900
N2—C8	1.464 (2)	C10—C11	1.515 (3)
N3—C10	1.494 (2)	C10—H10A	0.9900
N3—C13	1.495 (2)	C10—H10B	0.9900
N3—C9	1.495 (2)	C11—H11A	0.9900
C1—C2	1.389 (3)	C11—H11B	0.9900
C1—H1	0.9500	C12—C13	1.522 (3)
C2—C3	1.383 (3)	C12—H12A	0.9900
C2—H2	0.9500	C12—H12B	0.9900
C3—C4	1.395 (3)	C13—H13A	0.9900
C3—H3	0.9500	C13—H13B	0.9900
C4—C5	1.379 (3)	O2—H2A	0.837 (16)
C4—H4	0.9500	O2—H2B	0.831 (16)
			
N2—Cu1—N1	79.77 (6)	C6—C7—H7B	109.5
N2—Cu1—N3	84.20 (6)	H7A—C7—H7B	109.5
N1—Cu1—N3	163.90 (6)	C6—C7—H7C	109.5
N2—Cu1—Cl1	154.62 (5)	H7A—C7—H7C	109.5
N1—Cu1—Cl1	97.01 (4)	H7B—C7—H7C	109.5
N3—Cu1—Cl1	96.79 (4)	N2—C8—C9	106.53 (14)
N2—Cu1—Cl2	95.63 (5)	N2—C8—H8A	110.4
N1—Cu1—Cl2	88.97 (5)	C9—C8—H8A	110.4
N3—Cu1—Cl2	94.20 (4)	N2—C8—H8B	110.4
Cl1—Cu1—Cl2	109.544 (19)	C9—C8—H8B	110.4
C11—O1—C12	109.02 (14)	H8A—C8—H8B	108.6
C1—N1—C5	118.92 (16)	N3—C9—C8	110.42 (14)
C1—N1—Cu1	127.12 (12)	N3—C9—H9A	109.6
C5—N1—Cu1	113.06 (12)	C8—C9—H9A	109.6
C6—N2—C8	126.51 (15)	N3—C9—H9B	109.6
C6—N2—Cu1	118.51 (13)	C8—C9—H9B	109.6
C8—N2—Cu1	114.57 (11)	H9A—C9—H9B	108.1
C10—N3—C13	107.88 (14)	N3—C10—C11	113.72 (15)
C10—N3—C9	111.29 (14)	N3—C10—H10A	108.8
C13—N3—C9	112.19 (14)	C11—C10—H10A	108.8
C10—N3—Cu1	109.42 (11)	N3—C10—H10B	108.8
C13—N3—Cu1	112.81 (11)	C11—C10—H10B	108.8
C9—N3—Cu1	103.24 (10)	H10A—C10—H10B	107.7
N1—C1—C2	122.40 (17)	O1—C11—C10	110.79 (15)
N1—C1—H1	118.8	O1—C11—H11A	109.5
C2—C1—H1	118.8	C10—C11—H11A	109.5
C3—C2—C1	118.62 (17)	O1—C11—H11B	109.5
C3—C2—H2	120.7	C10—C11—H11B	109.5
C1—C2—H2	120.7	H11A—C11—H11B	108.1
C2—C3—C4	119.35 (17)	O1—C12—C13	111.41 (15)
C2—C3—H3	120.3	O1—C12—H12A	109.3
C4—C3—H3	120.3	C13—C12—H12A	109.3
C5—C4—C3	118.75 (17)	O1—C12—H12B	109.3
C5—C4—H4	120.6	C13—C12—H12B	109.3
C3—C4—H4	120.6	H12A—C12—H12B	108.0
N1—C5—C4	121.95 (16)	N3—C13—C12	112.83 (15)
N1—C5—C6	114.30 (15)	N3—C13—H13A	109.0
C4—C5—C6	123.69 (16)	C12—C13—H13A	109.0
N2—C6—C7	126.57 (17)	N3—C13—H13B	109.0
N2—C6—C5	113.71 (16)	C12—C13—H13B	109.0
C7—C6—C5	119.70 (16)	H13A—C13—H13B	107.8
C6—C7—H7A	109.5	H2A—O2—H2B	101 (2)

### Hydrogen-bond geometry (Å, °)

**Table:**

*D*—H···*A*	*D*—H	H···*A*	*D*···*A*	*D*—H···*A*
O2—H2A···Cl2^i^	0.84 (2)	2.35 (2)	3.1829 (16)	173 (2)
O2—H2B···Cl1	0.83 (2)	2.48 (2)	3.2841 (18)	164 (2)
C2—H2···O2^ii^	0.95	2.41	3.307 (2)	156
C3—H3···Cl2^iii^	0.95	2.82	3.619 (2)	142
C4—H4···O2^iv^	0.95	2.50	3.445 (2)	172
C7—H7A···Cl1^v^	0.98	2.68	3.6179 (19)	161
C8—H8A···O1^vi^	0.99	2.47	3.336 (2)	146
C10—H10B···Cl1	0.99	2.79	3.4496 (19)	124
C10—H10A···Cl2	0.99	2.71	3.3566 (19)	123

Symmetry codes: (i) *x*−1, *y*, *z*; (ii) −*x*+1, −*y*+1, −*z*; (iii) −*x*+2, −*y*+2, −*z*; (iv) −*x*+1, −*y*+2, −*z*; (v) *x*, *y*+1, *z*; (vi) −*x*+1/2, *y*+1/2, −*z*+1/2.
